# A Model of Traumatic Brain Injury Oligomerizes Tau in Cortical Organoids and Induces Clinically Relevant Pathologies that Synergize with *MAPT* Mutation

**DOI:** 10.1177/08977151251374286

**Published:** 2025-11-13

**Authors:** Shahrzad Shiravi, Alexandra Yufa, Paraskevi Papavasileiou, Steven Lotz, Dylan Murphy, Taylor Bertucci, Sally Temple, John D. Finan

**Affiliations:** ^1^ Department of Mechanical and Industrial Engineering, University of Illinois Chicago, Chicago, Illinois, USA.; ^2^ Department of Biomedical Engineering, University of Illinois Chicago, Chicago, Illinois, USA.; ^3^ Neural Stem Cell Institute, Albany, New York, USA.

**Keywords:** iPSC-derived organoids, *MAPT* mutation, neurodegeneration, tauopathy, tau oligomerization, traumatic brain injury

## Abstract

Traumatic brain injury (TBI) is the most important environmental risk factor for neurodegenerative disease. Tauopathy plays an important role in post-traumatic neurodegeneration. Human-induced pluripotent stem cell (hiPSC)-derived cortical organoids have exciting potential to reveal the influence of genotype on post-traumatic neurodegeneration because they permit manipulation of the genome in a human system. This study established an isogenic 3D cortical organoid model of TBI to investigate tau pathology and other clinically relevant injury phenotypes. Organoids generated from patient-derived hiPSC lines carrying the V337M or IVS10 + 16 Microtubule associated protein tau (*MAPT*) mutations and their clustered regularly interspaced short palindromic repeats (CRISPR)-corrected isogenic counterparts were subjected to consistent compressive injury. Mitochondrial dysfunction, cell viability, lactate dehydrogenase (LDH) release, neurofilament light chain (NF-L) release, tau hyperphosphorylation, and tau oligomerization were quantified using live-cell imaging, ELISA, Western blotting, and immunostaining post-injury. Pathology depended on the severity of the mechanical injury and the time since injury. The V337M mutation synergized with injury to exacerbate cell damage, increasing LDH release and reducing viability in 4- and 6-month-old organoids. Therefore, this model can reproduce gene-trauma interactions *in vitro,* so it has the capacity to answer important questions about why different patients have different outcomes after similar TBIs. *MAPT* mutation was not necessary for injury to induce tau hyperphosphorylation in 4-month-old organoids and both tau hyperphosphorylation and tau oligomerization in 6-month-old organoids. This capacity to induce advanced tau pathology in wild-type human organoids could have utility beyond the field of TBI research.

## Introduction

Traumatic brain injury (TBI) is a major global health challenge, affecting people of all ages. Each year, approximately 69 million people experience TBI globally.^1^ Fortunately, the vast majority of these injuries are mild (81%) or moderate (11%) rather than severe.^1^ However, even mild TBI increases the risk of neurodegenerative disorders such as Alzheimer’s disease^2^ and chronic traumatic encephalopathy.^3,4^ TBI outcomes are heterogeneous, with significant variability in recovery and long-term morbidity among individuals.^5^ Factors such as age,^6^ sex, and injury severity influence TBI outcomes.^7,8^ In addition, multiple lines of evidence indicate that TBI outcomes depend in part on genetic factors.

Genetic variants can increase TBI morbidity by either promoting risk-taking behaviors that lead to TBI or by diminishing the capacity of the brain to cope with and recover from TBI.^9^ In the latter class, the APOE4 allele^10^ and the val66met single-nucleotide polymorphism (SNP) in brain-derived neurotrophic factor (BDNF)^11,12^ were among the first variants to be studied because they were known to influence other neurological conditions. SNPs in the *ABCC8* gene, which encodes the sulfonylurea receptor 1, influence the development of cerebral edema after severe TBI.^13^ More recently, comprehensive observational studies of TBI patients incorporating genome sequencing have revealed that SNPs in other genes, such as Ankyrin Repeat and Kinase Domain containing I,^14^ interleukin-6,^15^ and B-cell lymphoma 2,^16^ influence TBI outcomes. Genome-wide association studies have identified other genetic variants, including some involved in immune response and neuronal repair, that influence recovery trajectories.^17,18^ Wang et al. identified 26 genes that changed their expression after TBI and found the microtubule associated protein tau (*MAPT*) mutation also influenced 16 of these 26 genes.^19^ In light of this evidence that TBI and *MAPT* affect similar gene networks, we hypothesized that *MAPT* mutation would exacerbate TBI pathology.

While clinical studies remain the gold standard for understanding how patient genotype influences TBI outcome and neurodegeneration after trauma,^20^ they also face formidable challenges. The heterogeneity of patients and injury events frustrates efforts to demonstrate statistically significant correlations.^21^ Diagnosing mild TBI remains particularly problematic due to reliance on subjective symptom reporting and the absence of definitive neuroimaging markers, resulting in potential misclassification.^22^ Therefore, an urgent need exists for a model of TBI that reduces heterogeneity in the experimental subjects and permits control of genotype while retaining human pathophysiology.^23^

*In vitro* models of TBI complement clinical studies because they standardize mechanical injury and isolate cells and/or tissues of interest. The first *in vitro* models of TBI used transection or fluid loading to injure rodent tissue cultures.^24^ Substrate stretch injury models emerged in 1995^25^ and became popular because they allowed the mechanical strain and strain rate to be tuned and matched to clinical conditions.^26–30^ *In vitro* models that permitted similar control with 3D cultures followed in 2006.^31^ Shortly thereafter, human-induced pluripotent stem cells (hiPSCs) arrived and revolutionized the study of neurological disease by creating the opportunity to reproduce human pathophysiology and manipulate the human genome in an *in vitro* model. HiPSC-derived neural cells have been integrated into 2D^30^ and 3D *in vitro* injury^32^ models to seize these opportunities in the context of TBI. Human cortical organoids have been loaded with ultrasound waves^33,34^ and compression^35,36^ to model blast-induced TBI and blunt-impact TBI, respectively. This study modeled TBI using compression injury of hiPSC-derived cortical organoids, so the genome and the mechanical injury could be controlled simultaneously.

Here, we used a novel protocol that generates highly consistent organoids.^37^ We used patient-derived hiPSCs from donors with the coding *MAPT* mutation V337M or the non-coding *MAPT* mutation IVS10 + 16 (both of which cause frontal temporal lobe dementia^38,39^) alongside clustered regularly interspaced short palindromic repeats (CRISPR)-corrected isogenic controls. Isogenic experiments compare two cell lines that differ only by the presence or absence of a single genetic variant so that any differences in experimental outcomes can be attributed to that variant with a high degree of confidence.^40^ The V337M *MAPT* mutation promoted phenotypes consistent with tauopathy in an isogenic study of human cortical organoids.^41^ Post-mortem neuropathology indicates that tauopathy plays an important role in premature neurodegeneration among individuals exposed to TBI.^42^ In this study, an existing model of TBI^35^ in human cortical organoids was refined to reduce the influence of variable organoid geometry on outcomes. The dependence of outcomes on injury severity and time since injury was measured, and these results informed the design of an isogenic experiment to test the hypothesis that the *MAPT* mutation exacerbates pathology after injury in a human system. Outcomes included markers of mitochondrial depolarization, an important early pathology of neurotrauma,^43^ and neurofilament light chain (NF-L) release, which is elevated in humans after TBI.^44^ Tau pathology was quantified by measuring tau hyperphosphorylation, which has been induced in organoids with trauma before,^44^ and tau oligomerization, which has only previously been induced in organoids using multiple genetic manipulations.^45^

## Methods

### Cell culture

Multiple iPSC lines were used in this study. Eight isogenic lines were obtained from the Tau Consortium (Supplementary Table S1).^46^ Cells were maintained in 6-well plates coated with growth factor-reduced Cultrex at 37°C and 5% CO_2_. The cultures were fed two to three times a week using mTeSR1 medium with a sustained-release formulation of native fibroblast growth factor (FGF2) in an FGF2DISC (StemCultures; DSC500-48). The FGF2DISC slowly releases FGF2 and maintains a 10 ng/mL level of FGF2 in the culture medium, which counteracts the normal short half-life of FGF2 in culture medium.^37^ One FGF2DISC was added to one well of a 6-well plate with 2 mL of mTeSR1 medium for approximately a week. The medium, but not the FGF2DISC, was replaced every 2–3 days based on culture confluency. In either culture method, hiPSC cultures were clump-passaged using ReLeSR about once a week. Cells were not allowed to grow past 80% confluency. To prepare for organoid production, hiPSC colonies were single-cell passaged using TrypLE Express. The WTC-11(Coriell, GM25256) line was used to identify the injury severity that induced pathology and the time course of that pathology. These cells were cultured on Matrigel (Sigma-Aldrich, CLS354277) coated plates and maintained in mTeSR plus basal medium (Stem Cell Technologies, 100-0276) plus 1% penicillin-streptomycin (Thermofisher, 15140122) for 5–6 days with daily media changes to reach 75–80% confluency. All lines were negative for mycoplasma and karyotypically normal by G-banded Karyotype (WiCell Research Institute, Inc.). HiPSC pluripotency was confirmed according to ISSCR guidelines by immunofluorescent labeling of Octamer-binding transcription factor 4 (OCT4) and Stage-Specific Embryonic Antigen-4 (SSEA4) (Supplementary Fig. S4) or similar canonical pluripotency markers.

### Organoid production

Organoids derived from the isogenic cell lines were generated at the NeuraCell core facility (Neural Stem Cell Institute, NY, USA). These organoids were generated in 96-slitwell plates followed the methods described previously.^37^ In brief, when hiPSC cultures reached about 70–80% confluency, hiPSCs were single-cell harvested using TrypLE Express. Cells were counted manually with a hemocytometer and resuspended at approximately 1 million cells/mL in mTeSR1 with 10 µM ROCK inhibitor Y-27632 (Tocris). Each hiPSC line was plated into a separate 96-slitwell plate (S-bio, MS9096SZ) at a density of 10,000 cells per well to achieve day 1 hiPSC spheroids 350–500 µm in diameter. The next day, the plate was washed twice, and the medium was replaced with 14 mL of differentiation Medium A: E6 base medium supplemented with 2.5 µM dorsomorphin (DM), 10 or 20 µM SB431542 (cell-line dependent level as described^37^), and 2.5 µM XAV939. On the sixth day, the medium was changed to Medium B: neural medium (NM) base with 20 ng/mL epidermal growth factor (EGF) plus 20 ng/mL FGF2. The medium was changed daily for 10 days, followed by 9 days of feeding three times per week. On day 25, the medium was replaced with Medium C, which consisted of NM supplemented with 20 ng/mL BDNF and 20 ng/mL neurotrophic factor 3 (NT3), with media changes three times per week. From day 43 onward, the medium was changed three times per week with Medium D (NM base with no added growth factors) with 15–20 mL per slit-well plate (approximately 75% medium changes). Successful forebrain patterning was achieved for all 8 lines, determined by qPCR at 20-days for the cortical progenitor markers PAX6 and FOXG1 and by further analysis of expression of cortical neuron markers CTP2 and TBR1 in 2-month organoid cryosections by immunohistochemistry (IHC).

WTC-11 organoids were made using Dorsal Forebrain Organoid Kits (StemCell Technologies, 08620), following a previously published protocol.^47^ Briefly, hiPSCs were released, centrifuged, and transferred to Aggrewell 800 microwell culture plates (StemCell Technologies, 34815) at a density of 10,000 cells per microwell, where they spontaneously aggregated. Aggregates were treated with dual Suppressor of Mothers against Decapentaplegic (SMAD) inhibition for 6 days and were subject to partial daily media changes. On day 7, aggregates were transferred to ultra-low-attachment 6-well plates (30–40 organoids per well) and supplied with serum-free medium with FGF2 and EGF until day 25. Then, they were moved to media containing BDNF and NT3 until day 43, after which they were maintained in media without growth factors. A mixture of four small molecules—GSK2879552 (Selleck Chemicals, S7796), EPZ5676 (Selleck Chemicals, S7062), Bay K 8644 (Selleck Chemicals, S7924) and NMDA (Selleck Chemicals, S7072), all at a concentration of 1 µM was added to the medium to enhance neuron maturation^48^ in cortical organoids from day 15 until the day of experiments. Microtubule-associated protein 2 (MAP2), S100 calcium-binding protein B (S100B), and SRY-box 2 (SOX2) expression at day 60 confirmed the presence of neurons, immature astrocytes, and neural progenitor cells, respectively, in these cultures. By day 180, glial fibrillary acidic protein (GFAP) expression indicated that astrocytes had become more mature (Supplementary Fig. S2).

### Fixation and frozen sectioning of isogenic organoids

Organoids were fixed in cold 4% paraformaldehyde and placed at 4°C overnight. They were then rinsed three times in 3 mL phosphate-buffered saline (PBS) and allowed to sink in 30% sucrose in PBS. The organoids were embedded using Optimal Cutting Temperature (OCT) compound (Tissue Tek 4583) in cryomolds (Seal N Freeze), frozen, and stored at −80°C. Organoids were sectioned using a cryostat. Sections were placed on microscope glass slides, dried overnight at room temperature, and stored at −20°C for future immunofluorescence.

### Immunofluorescent staining of organoid sections

Slides were thawed and brought to room temperature (RT). The sections were rehydrated with PBS, blocked, permeabilized, and immunostained with a selection of primary antibodies: MAP2 (1:1000, synaptic systems 188004 Lot# 7–54), CTIP2 (1:500 Abcam ab18465 Lot#2101050859), S100B (Sigma-Aldrich, S2532, 1:100), SOX2 (Sigma-Aldrich, AB5603, 1:100), T22 (Sigma- Aldrich, ABN454, 1:500) and CP13 (Gift from Peter Davies). Primary antibodies were incubated overnight at 4°C, washed three times with PBS, and then incubated with corresponding Alexa Fluor conjugated secondary antibodies: 1:300 AF goat anti-guinea pig 647 (Invitrogen A21450 Lot#2420694), 1:1000 AF goat anti-rat 488 (Invitrogen A11006 Lot#2480078), AF 647-Goat anti Mouse (Jackson, 115-605-003, 1:500), Alexa 568-goat anti rabbit (Thermofisher, A-11011), and AF 647 goat anti rabbit (Jackson, 111-605-144, 1:500) for 1 h at RT. Sections were washed three times with PBS again and then coverslipped and imaged using fluorescence microscopy.

### Western blotting

Organoids were lysed in 1X radioimmunoprecipitation assay (RIPA) buffer with added protease (1:50) and phosphatase inhibitors (1:10). 50–75 μL per organoid of RIPA solution was added to each sample. Samples were sonicated for 10 min with a 30-second on and off cycle at 4°C. Samples were then further dissociated as needed after sonication using a pipette. A detergent compatible (DC) assay was used to determine protein concentration, and raw lysates were diluted in Laemmli SDS-sample buffer at ∼1 μg/μL. 10 µg of protein was separated using a 10% BisAcrylamide gel in an ice bucket for about 2.5 h. Proteins were transferred to a nitrocellulose membrane using a semi-wet transfer system at 25 V and 2.5 A for 20 min. Membranes were blocked in Intercept® Blocking Buffer tris-buffered saline (TBS) (cat. 927–60001) at a 1:1 dilution with TBS for 2 h at room temperature with gentle agitation followed by incubation of primary antibodies Total Tau (D5D8N, cat. 4394S), AT8 (cat. MN1020), Actin (D18C11, cat. 8456S), PHF1 (gifted by Peter Davies), and CP13 (gifted by Peter Davies) diluted in Intercept® Blocking Buffer TBS at a 1:1 dilution with TBST (0.2% Tween®) overnight at 4°C. Washes were done in TBST (0.2% Tween) with vigorous agitation, 5 min per wash, for a total of four washes. The blots were incubated with their respective fluorescent secondary antibodies (1:20,000) and/or chemiluminescent secondary antibodies (1:2,500) for 1 h at room temperature, washed in TBST (0.2% Tween), with vigorous agitation, 5 min per wash, for a total of four washes, with a final fifth wash in TBS. The membranes were placed in diH_2_O and imaged in the Odyssey® M Imaging System, with quantification done using the Empiria Studio program, normalizing to actin.

### Organoid RNA isolation for qPCR

Total RNA was isolated from organoids with the Trizol RNA Clean and Concentrator Kit. RNA concentrations were measured on a Nanodrop, and cDNA was generated using the High-Capacity cDNA Reverse Transcription Kit. Real-time quantitative PCR was performed using Power SYBR Green Master Mix. Glyceraldehyde-3-phosphate dehydrogenase(GAPDH) was used for the housekeeping gene. Forward and reverse primers are listed in Supplementary Table S2.

### Injury experiments

Organoids were injured using a custom-built injury device (Fig. 1). The linear actuator and feedback control of the device were as previously reported,^35^ but the indentation apparatus was reconfigured to injure one organoid at a time so that the amplitude of the compressive motion could be adapted to the height of each organoid. The injury device consisted of a flat, stationary platform and a vertically translating stage (Fig. 1A, B). A flat-ended, stainless-steel indenter was mounted on the stage with a pair of right-angle brackets. A LA43-67-000A voice coil (BEI Kimco) controlled by an Xenus XTL-230–40 servo drive (Copley Controls) drove the vertical motion of the stage. The servo drive used closed-loop feedback from a T1031-30A linear quadrature encoder (Renishaw PLC) to monitor stage position. During an injury procedure, organoids were placed under the indenter in an 8-well chamber slide (Ibid., 80801) on an XY stage that permitted fine adjustment of position in the horizontal plane. Before any organoids were placed in the chamber slide, the indenter was incrementally brought into contact with the bottom of the chamber, and that position was recorded. Then, the indenter was raised, an organoid was placed in the chamber, and the indenter was incrementally brought into contact with the organoid to initialize the injury procedure. The contact position for the chamber bottom was subtracted from the contact position for the vertex of the organoid to compute the organoid height. Then, organoids were dynamically compressed by moving the indenter down and up in a period of approximately 30 msec through a distance equal to a fraction of the organoid height (henceforth referred to as the compression ratio) that depended on the desired injury severity. To maintain sterility, the indenter was autoclaved beforehand, and the entire procedure was executed in a biosafety cabinet. Each organoid was out of the incubator for less than 10 min. After the experiments were complete, an injury procedure was imaged in the vertical plane at 500 frames per second using a Photron FastCam Mini WX100 camera to create images illustrating the procedure (see Fig. 1C).

**FIG. 1.**
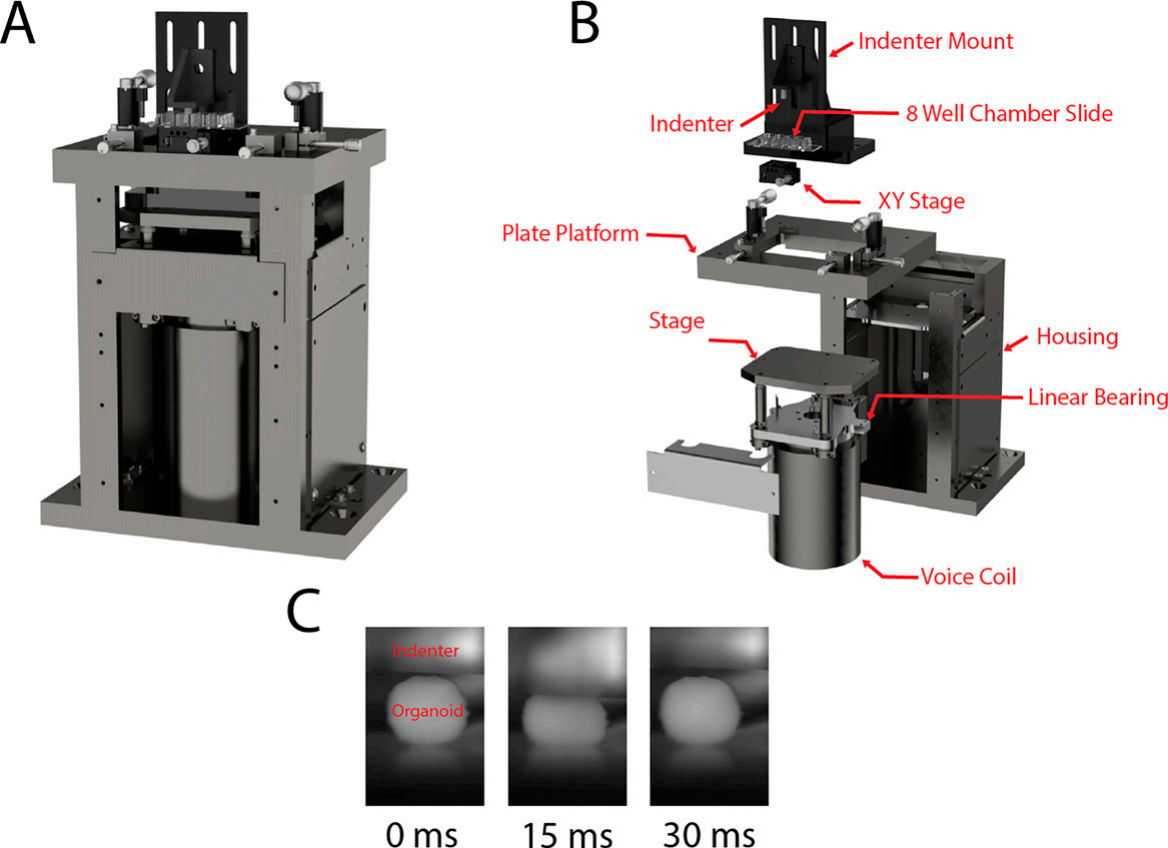
Injury machine **(A)** Custom-built injury device. **(B)** Exploded view of custom-built injury device components. **(C)** Stills from high-speed video in the vertical plane of an organoid during an injury experiment.

### Microscopy and image analysis

Three-dimensional stacks of live fluorescent images were acquired using an Olympus FV3000 confocal microscope and a 4X objective. All images in each experiment were acquired with the same microscope settings. Maximum intensity projections of the 3D images were created with ImageJ. Fluorescent intensity was quantified by segmenting and averaging fluorescent images in CellProfiler 4.0 software.^49^

### Live staining

To measure cell viability and mitochondrial membrane potential, organoids were incubated with Calcein AM (Thermofisher, C1430) and tetramethylrhodamine (TMRM, Millipore Sigma, T5428) at 37°C for 60 min at 1 µM and 100 nM concentrations, respectively. Organoids were washed with Dulbecco’s PBS three times after incubation and then imaged in cell culture media.

### Measurement of lactate dehydrogenase (LDH)

50 µL of supernatant was collected from each sample immediately before injury and at the specified time point. ΔLDH was calculated by subtracting the pre-injury absorbance value from the post-injury value. Assays were performed using an LDH Cytotoxicity Kit (Promega, G1780) according to the manufacturer’s instructions. Briefly, 50 µL of CytoTox 96 reagent was added to each sample and incubated for 30 min at room temperature, followed by the addition of 50 µL of Stop Solution. LDH absorbance was measured at a wavelength of 492 nm using a BMG Optima microplate reader. Organoids were imaged using brightfield microscopy before injury. The area and radius were measured in ImageJ and used to estimate the organoid volume, based on the approximation of the 3D organoid geometry as a sphere. ΔLDH values were normalized to organoid volume.

### Measurement of neurofilament light chain

NF-L was measured using a human NF-L ELISA kit from Novus Biologicals (NBP2-81184) according to the manufacturer’s instructions. Briefly, 100 µL of each sample was loaded into the provided 96-well ELISA plate. After incubation, the samples and standards were aspirated, and 100 µL of biotinylated detection antibody was added. Then, the plate was incubated at 37°C for 1 h and washed five times with washing buffer. Next, 100 µL of horseradish peroxidase conjugate was added and incubated at 37°C for 30 min, followed by aspiration and another five washes. Finally, 90 µL of substrate reagent was added and incubated at 37°C for 15 min, followed by the addition of 50 µL of stop solution. The absorbance in each well was measured at 540 nm wavelength using a BMG Optima plate reader. Background signal was corrected by subtracting the average absorbance of the zero standard (sample diluent only). The concentration of NF-L was determined using a standard curve.

### Statistical analysis

Statistical significance was determined using analysis of variance (ANOVA), or repeated measures ANOVA as appropriate to the experimental design, followed by Tukey’s multiple comparisons test. All statistical analyses were performed using IBM SPSS software.

## Results

Organoids supplied from Neuracell were provided with a certificate of analysis demonstrating that the growth curves from eight distinct lines, averaged across 3–5 organoids per condition on three independent plates, demonstrated consistent growth trajectories across all lines (Supplementary Fig. S1A). A summary of the organoid lines and experimental replicates is provided in Supplementary Figure S1B. Gene expression analysis by qPCR at 20 days of differentiation confirmed that all eight lines met quality control (QC) criteria for forebrain identity, as indicated by robust expression of PAX6 and FOXG1 above the QC threshold (Supplementary Fig. S1C). The negative QC marker COL1A2 confirmed the absence of mesoendodermal off-target cell populations. Each line was evaluated with three organoids pooled per plate across three independent plates. Immunohistochemistry analysis of 2-month-old organoid sections stained for MAP2 (pan-neuronal marker) and CTIP2 (deep-layer cortical marker) further validated forebrain identity and cortical organization in all lines (Supplementary Fig. S1D). These results confirm that all eight lines passed QC checkpoints for use in downstream experiments.

### Mitochondrial pathology correlated with injury severity and recovered over time

The amount of deformation applied to the culture was quantified using the compression ratio, which was defined as the amplitude of the motion of the indenter divided by the initial height of the organoid. The magnitude of the injury phenotype increased as the compression ratio increased. Specifically, 24 h post-injury, mitochondrial membrane potential (quantified using TMRM live cell staining) declined with increasing compression ratio (Fig. 2A, B). ΔLDH trended upwards as compression ratio increased, although this trend did not become statistically significant until the most severe injury condition, 45% compression (see Fig. 2C). The LDH signal at 45% compression was more than double that at 35%, suggesting a partial threshold response in this outcome in contrast to the approximately linear response observed for TMRM intensity. For a follow-up experiment looking at the evolution of the injury phenotype over time, the compression level was set at 30%. This value was chosen because it is near the middle of the approximately linear decline in TMRM intensity with increasing compression and TMRM intensity was the primary outcome for experimental design. Mitochondrial membrane potential recovered to normal levels within 7 days of injury at 30% compression (Fig. 2D, E). ΔLDH levels rose until 48 h post-injury and remained unchanged between 48 and 72 h (Fig. 2F). Due to the required media change at 72 h, supernatant samples were not collected 7 days after injury so no ΔLDH data is available at this timepoint.

**FIG. 2.**
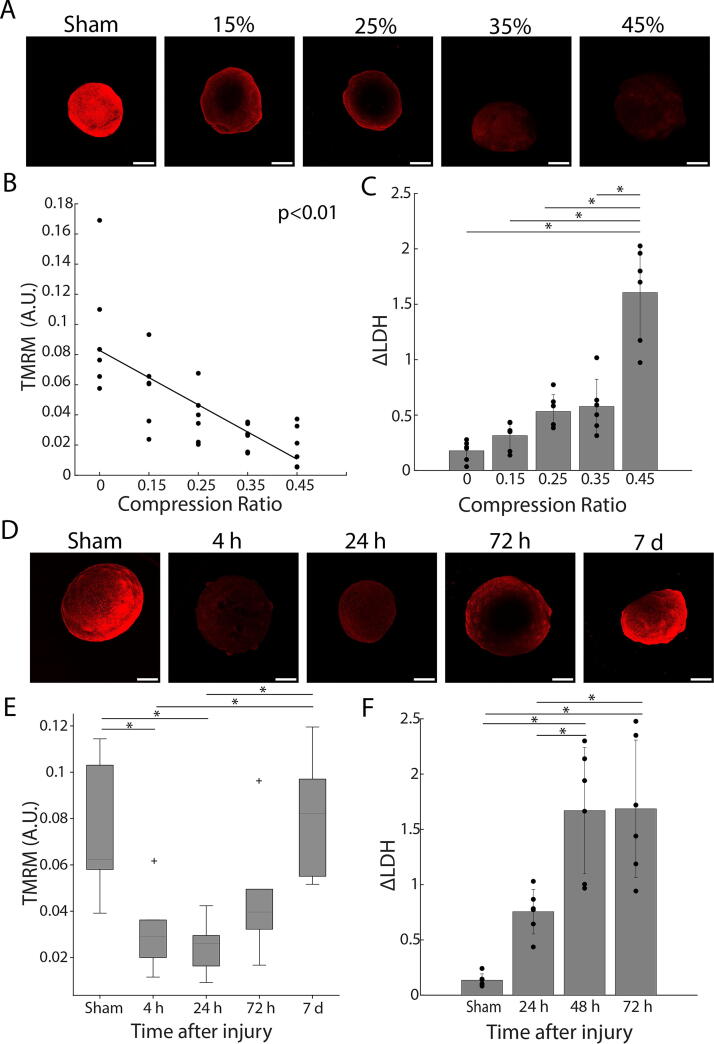
Effects of injury severity on mitochondrial membrane potential and LDH release. **(A)** Representative images of TMRM fluorescence 24 h after injury in 79–80-day old WTC-11 organoids subjected to injury of various severities (scale bar = 400 µm, *n* = 6 organoids). **(B)** TMRM fluorescence intensity 24 h after injury (line represents linear regression, *p* < 0.01, A.U., arbitrary units). **(C)** ΔLDH intensity across injury of various severities 24 h after injury (*p < 0.01, ANOVA followed by Tukey’s test). **(D)** Representative images of TMRM fluorescence in 79–80-day organoids injured with a 30% compression ratio at various times post-injury (scale bar = 500 µm, *n* = 6 organoids). **(E)** Time course of TMRM fluorescence intensity following injury with a compression ratio of 30% (*p* < 0.001, ANOVA followed by Tukey’s tests, grey boxes represent inter-quartile range [25th–75th percentiles], whiskers represent 5th and 95th percentiles, + symbol represents data points outside of the 5th and 95th percentile range). **(F)** ΔLDH at various times post-injury after injury with a compression ratio of 30% (*p < 0.01, ANOVA followed by Tukey’s tests). ANOVA, analysis of variance; LDH, lactate dehydrogenase; TMRM, tetramethylrhodamine.

### V337M mutation amplified injury-induced pathology

The effect of injury on mitochondrial membrane potential, ΔLDH, and cell viability was evaluated in 4-month-old cortical organoids. Organoids were generated from 8 lines: 2 lines derived from 2 patients carrying the V337M mutation in the *MAPT* gene (GIH 6 and GIH 7), along with corresponding isogenic control lines in which the mutations had been corrected, and 2 lines derived from 2 patients carrying the IVS10 + 16 mutation in *MAPT* (GIH 36 and GIH 178), along with isogenic controls in which the mutation had been corrected. Note that V337M is a coding mutation and IVS10 + 16 is a non-coding mutation. Several phenotypes indicated that compression injured organoids. At 48 h post-injury, TMRM intensity and calcein AM intensity both declined as injury severity increased (ANOVA followed by Tukey’s test, *p* < 0.05, Fig. 3A, B). ΔLDH increased with increasing injury severity (ANOVA followed by Tukey’s tests, *p* < 0.05, Fig. 3C).

**FIG. 3.**
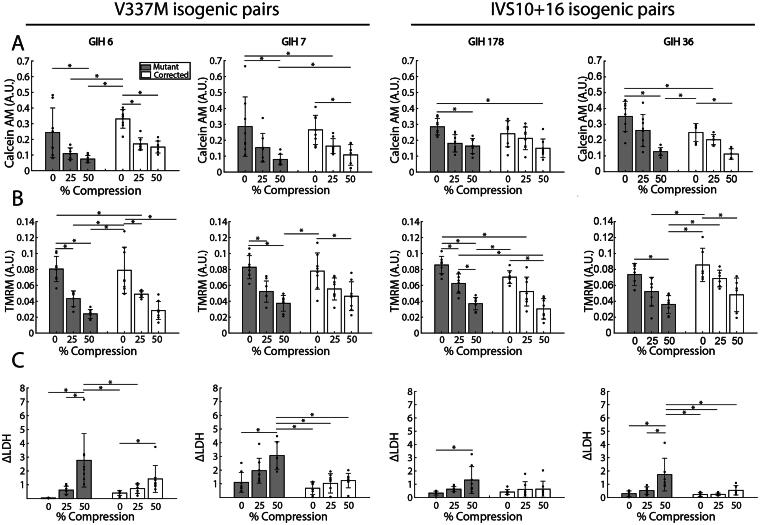
The effect of injury on 4-month-old organoids with a *MAPT* mutation and their respective isogenic controls. **(A)** Mean Calcein AM fluorescence intensity at 0%, 25%, and 50% compression across all cell lines 48 h after injury (ANOVA followed by Tukey’s test, *=*p* < 0.05, *n* = 8 organoids). **(B)** Mean TMRM fluorescence intensity at 0%, 25%, and 50% compression across all cell lines 48 h after injury (ANOVA followed by Tukey’s test, *p < 0.05, *n* = 8 organoids). **(C)** ΔLDH 48 h after injury (ANOVA followed by Tukey’s test, *p < 0.05, *n* = 8 organoids).

A statistically significant mutation-injury interaction occurred in organoids carrying the V337M mutation and their isogenic corrected counterparts (ANOVA followed by Tukey’s tests, *p* < 0.05). Specifically, significant interactions were observed for ΔLDH (η^2^ = 0.138, *p* = 0.002), calcein AM (η^2^ = 0.136, *p* = 0.002), and TMRM (η^2^ = 0.121, *p* < 0.001), indicating that this mutation amplifies the effects of injury on cell damage, viability, and mitochondrial membrane potential, respectively. In contrast, no such statistically significant interactions were found for organoids carrying the IVS10 + 16 mutation and their isogenic counterparts.

The mutations affected viability and LDH release but not mitochondrial membrane potential. There was a significant main effect of the IVS10 + 16 and V337M mutations on calcein AM and ΔLDH (ANOVA, *p* < 0.05) when cell lines with each mutation were pooled. However, no main effect of mutation was found in TMRM, suggesting that mitochondrial membrane potential was unaffected by the mutation alone.

The effect of the donor on the phenotypes measured was slight. No statistically significant effect occurred for the IVS10 + 16 donor lines. For the V337M donor lines, the effect was not statistically significant for Calcein AM or TMRM intensity but was statistically significant for ΔLDH, where it accounted for 0.9% of the variance (ANOVA, *p* = 0.005, η^2^ = 0.009).

### Injury increased tau hyperphosphorylation in both MAPT mutant and corrected 4-month-old organoids

Injury increased tau hyperphosphorylation 48 h post-injury in organoids generated from patients with tauopathy-associated mutations, as measured by both CP13/total tau and PHF1/total tau (repeated measures ANOVA, *p* < 0.05). This effect persisted when the tau mutation was corrected (Fig. 4A, B). There was no statistically significant interaction between the effects of injury and mutation on tau hyperphosphorylation (repeated measures ANOVA, *p* = 0.2 and *p* = 0.8 for PHF1 and CP13, respectively). Injury had no statistically significant effect on total tau (Fig. 4C). Immunostaining with the MAP2 and T22 antibodies, which stain neurons and oligomeric forms of tau, respectively, did not reveal changes in neuron survival or tau oligomerization due to injury or mutation (Fig. 4D, E). Therefore, the changes observed in tau (Fig. 4A–C) occurred in the context of approximately constant populations of neurons and quantities of tau oligomers.

**FIG. 4.**
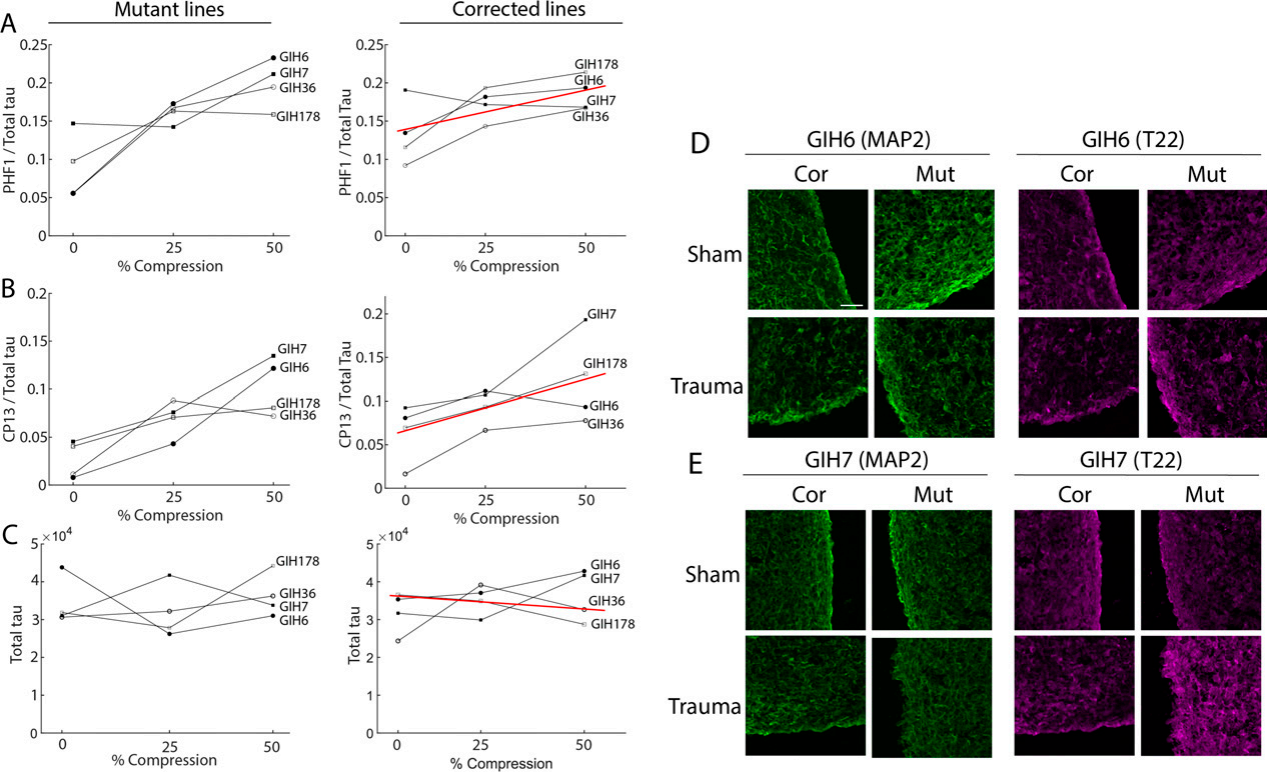
The effect of injury on tau phosphorylation in 4-month-old isogenic organoids. **(A)** Ratio of PHF1 to total tau in 4-month-old mutant and corrected cell lines 48 h after injury of various severities according to western blot quantification (each data point represents the value measured from three pooled organoids, the red line represents a statistically significant linear regression with *p* < 0.05), applied to the corrected lines, which were treated as four independent samples. The mutant organoids were not treated as four independent samples for linear regression because two of them shared one relevant mutation and two of them shared another relevant mutation. **(B)** Ratio of CP13 to total tau in 4-month-old mutant and corrected cell lines 48 h after injury of various severities according to western blot quantification (each data point represents the value measured from three pooled organoids; line represents linear regression with *p* < 0.05). **(C)** Total tau in 4-month-old mutant and corrected cell lines 48 h after injury of various severities according to western blot quantification (arbitrary units, each data point represents the value measured from three pooled organoids, line represents linear regression with *p* = 0.4). **(D)** MAP2 and T22 immunostaining in GIH6 cell lines carrying the V337M mutation **(E)** MAP2 and T22 immunostaining in GIH7 cell lines carrying the V337M mutation (representative of images from 3 different organoids, scale bar = 30 µm).

### V337M mutation and injury exert a synergistic effect on pathology in 6-month-old organoids

After observing the injury-induced tau pathology in 4-month-old organoids, a follow-up study with 50% compression was done at 6 months. The selection of this time point was motivated by prior findings that tau pathology becomes more pronounced as organoids carrying the V337M mutation age.^40,45^ Injury and V337M mutation synergized strongly in 6-month-old organoids carrying the V337M mutation (GIH 6 and GIH 7). Lines isolating the IVS10 + 16 mutation (GIH 36 and GIH 178) were not included in this experiment, as there was no significant gene-trauma interaction seen in the outcomes in these lines at 4 months. In 6-month-old organoids, as in 4-month-old organoids, TMRM and calcein AM fluorescence intensities declined 48 h after injury across GIH 6 and GIH 7 lines (Fig. 5A, B). Both NF-L release and ΔLDH increased significantly in the mutant groups (ANOVA followed by Tukey’s tests, *p* < 0.05). In the corrected cell lines, while an upward trend was observed, the increase was not statistically significant (Fig. 5C, D). A three-way ANOVA on injury, mutation, and parent cell line revealed a statistically significant main effect of all three factors and a significant interaction between mutation and injury (*p* < 0.05) for each of the four outcomes presented in Figure 6. While the main effect of parent line was statistically significant, the magnitude of this effect was relatively small (η^2^ < 0.1). Notably, 6-month-old *MAPT* mutation organoids showed more pronounced injury phenotypes compared to corresponding corrected cell lines. Significant interactions were observed for LDH (η^2^ = 0.8, *p* < 0.001), calcein AM (η^2^ = 0.181, *p* < 0.001), TMRM (η^2^ = 0.262, *p* < 0.001), and NF-L (η^2^ = 0.362, *p* < 0.001).

**FIG. 5.**
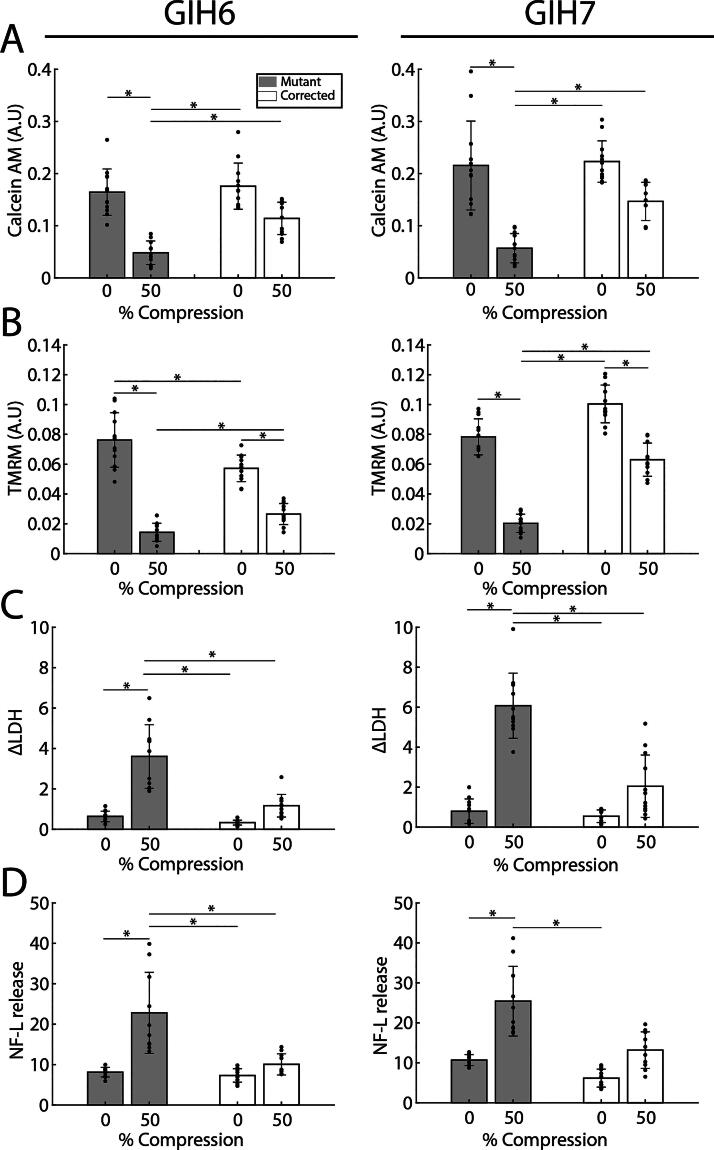
The effect of injury on 6-month-old isogenic organoids. **(A)** Mean Calcein AM fluorescence intensity across all cell lines 48 h after injury (ANOVA followed by Tukey’s tests, *p* < 0.05, *n* = 10 organoids). **(B)** Mean TMRM fluorescence intensity across all cell lines 48 h after injury (ANOVA followed by Tukey’s tests, *p* < 0.05, *n* = 10 organoids). **(C)** ΔLDH (ANOVA followed by Tukey’s test, *p* < 0.05, *n* = 10 organoids). **(D)** NF-L release across all cell lines 48 h after injury (ANOVA followed by Tukey’s test, *p* < 0.05, *n* = 10 organoids). NF-L, neurofilament light chain.

**FIG. 6.**
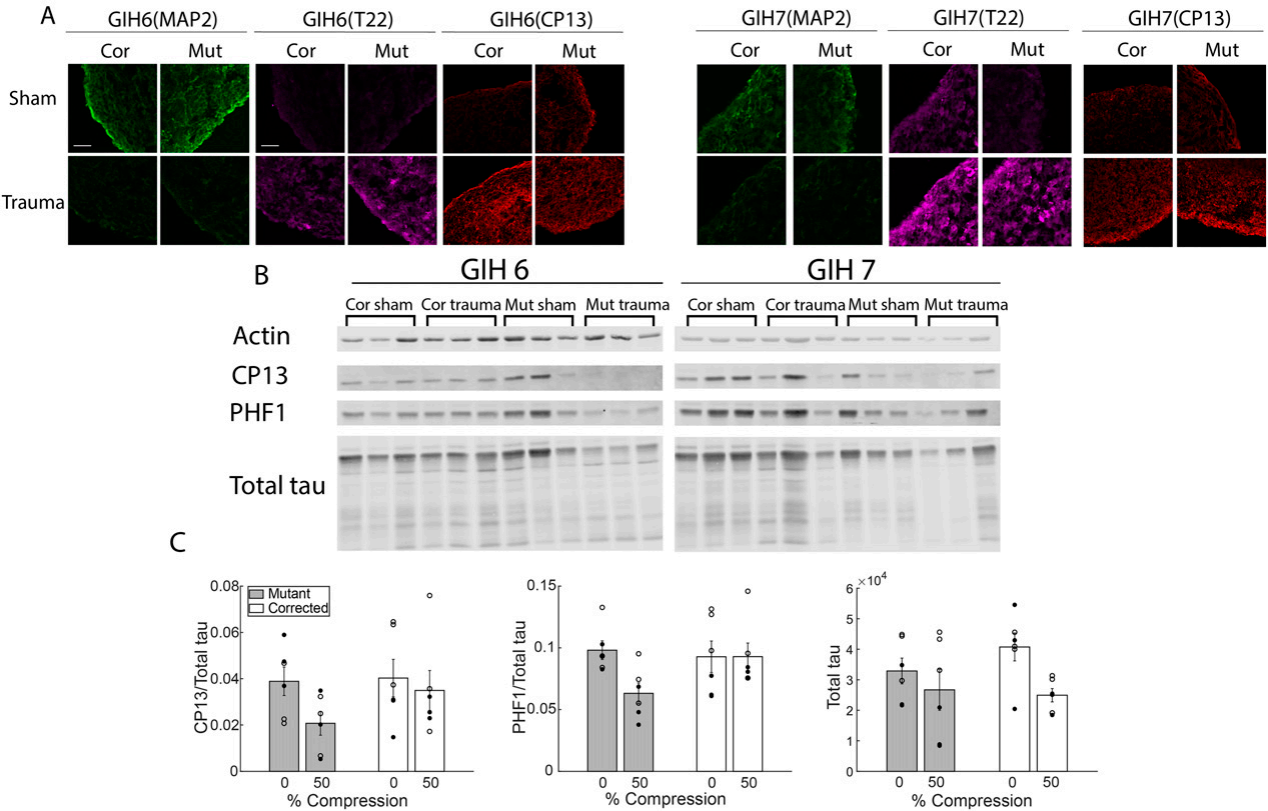
Injury effect on tau pathology in 6-month-old organoids. **(A)** MAP2, T22, and CP13 immunostaining in GIH 6 and GIH 7 lines (representative of images from three different organoids, scale bar = 30 µm). **(B)** Western blots for phosphorylated and total tau markers. **(C)** Quantification of CP13 to total tau ratio, PHF1 to total tau ratio, and total tau (arbitrary units). GIH 6 cell lines = solid circles, GIH 7 cell lines = hollow circles, *n* = 3 organoids per line per condition.

### Injury increased tau hyperphosphorylation and oligomerization in 6-month-old organoids

Injury increased tau hyperphosphorylation and oligomerization in 6-month-old organoids, as evidenced by increased CP13 staining and increased T22 staining (Fig. 6A), respectively. Despite the increase in CP13 immunofluorescence, the ratio of CP13 to total tau in western blots decreased after trauma, as did the ratio of PHF1 to total tau (Fig. 6B). Injury had a significant main effect on the ratios of CP13 and PHF1 to total tau, as well as on total tau levels (Fig. 6C, two-way ANOVA, *p* < 0.05). A significant interaction was observed between injury and mutation for PHF1/total tau (2-way ANOVA, *p* < 0.05). In combination, these results imply that, after injury, hyperphosphorylated tau was drawn into oligomers that were too heavy to appear in the same bands on the western blot as hyperphosphorylated tau monomers, as has been observed previously.^50^ MAP2 staining decreased after injury. The decline in MAP2 coincided with increased NF-L release (Fig. 5D), decreased Calcein AM (Fig. 5A), and increased ΔLDH (Fig. 5C). Taken together, these data suggest a decline in the neuron population after injury, which may explain the observed decline in total tau (Fig. 6C).

## Discussion

Our central hypothesis was that, in this organoid model of TBI, phenotypes relevant to acute morbidity and long-term neurodegeneration risk would be induced by injury in a genotype-dependent manner. The specific genotypes we investigated were the V337M and IVS10 + 16 mutations in the *MAPT* gene, both of which cause frontal temporal lobe dementia.^38,39^ The phenotypes measured included some directly related to tauopathy, such as tau hyperphosphorylation and tau oligomerization, and others that play an important role in TBI pathology but do not directly relate to tauopathy, such as mitochondrial membrane potential and NF-L release. Injury affected all these phenotypes in at least some of the scenarios tested. Surprisingly, the effect of injury never synergized with the effect of *MAPT* mutation for phenotypes related to tauopathy, even though the two effects synergized in several cases for phenotypes unrelated to tauopathy. *MAPT* mutation made organoids more vulnerable to injury, but was not required for injury to cause tauopathy. The fact that tau became hyperphosphorylated and oligomerized after injury in wild-type organoids indicates that cortical organoids are a promising human system in which to study post-traumatic tauopathy. Shifting the focus from TBI to tauopathy, this *in vitro* injury model induced advanced tau pathology without genetic manipulation in a human pre-clinical system. It could therefore be useful in phenotype-driven screening for tauopathy interventions. The specifics of the injury phenotype depended on the age of the organoids at the time of injury.

The response of 4-month-old organoids to injury resembled that of 6-month-old organoids in some ways, but the two age groups also differed in important ways. Overall, older organoids exhibited more influence of *MAPT* mutations and were capable of more advanced tauopathy, which is consistent with earlier studies of cortical organoids with *MAPT* mutations.^40,45^ In both age groups, injury damaged and killed cells (Fig. 3A, 5A) and depolarized the mitochondrial membrane (Fig. 3B, 5B). In addition, at 6 months, it triggered the release of NF-L (Fig. 5D), a phenomenon that also occurs in human subjects after TBI.^51^ In 4-month-old isogenic organoids, the coding mutation (V337M) in *MAPT* exacerbated the changes in cell viability and LDH release but did not exacerbate changes in mitochondrial membrane potential (Fig. 3), while the non-coding mutation (IVS10 + 16) had no statistically significant effect. This difference may reflect differences in the effects of these two mutations on tau proteins. V337M makes tau more prone to hyperphosphorylation.^52^ This effect appears to stress cells and diminish their capacity to cope with the additional stress of trauma. IVS10 + 16 alters the splicing of tau, thereby increasing the ratio of four repeat (4R) tau to three repeat (3R) tau.^53^ 4R tau binds to tubulin three times more strongly than 3R tau^54^ so it stabilizes microtubules more effectively.^55^ Microtubule fracture is an important initiating pathology in neurotrauma^56^ and stabilizing microtubules with taxol protects them against trauma *in vitro.*^57^ Therefore, IVS10 + 16 may exert a protective effect against trauma pathology that partially compensates for its tendency to promote tauopathy, and these competing effects may explain why IVS10 + 16 did not synergize with trauma while V337M did.

In 6-month-old organoids, the synergy between injury and the V337M mutation was more pronounced for all the phenotypes mentioned above. In 4-month-old organoids, injury increased tau hyperphosphorylation but did not cause obvious tau oligomerization (Fig. 4). In 6-month-old organoids, injury increased tau hyperphosphorylation and oligomerization in immunofluorescent images but decreased the tau hyperphosphorylation signal in western blots (Fig. 6). Taken together, these results suggest that some of the tau that became hyperphosphorylated after injury assembled into oligomers that were too heavy to reach the band of hyperphosphorylated tau monomers in the western blot. This pattern has been reported before. A recent preprint describes a mouse model of tauopathy in which phosphorylated tau (CP13, PHF1) declined in soluble fractions despite an increase in immunostaining when T22-positive tau oligomers accumulated in affected brain regions.^50^ The authors concluded that insoluble aggregates can sequester phosphorylated tau, making it less visible in western blots even though it remains visible in histological staining. Biofidelic oligomeric tau pathology matters because tauopathy plays an important role in clinical neurodegeneration after TBI.^58^ The clinical relevance of this phenotype is enhanced by the fact that it was induced using a clinically realistic mechanical insult.

To maximize the translational utility of our model, we designed the mechanical insult to mimic the mechanical insult experienced by cortical tissue during a clinical blunt impact TBI event. Brain tissue is effectively incompressible, so any change in its shape involves compression, tension, and shear simultaneously on different planes. Our injury model induces compressive strain on the vertical plane, tensile strain on the horizontal plane, and shear strain on diagonal planes. The parameter used most frequently to quantify deformation in TBI biomechanics is the maximum principal strain (MPS).^59,60^ The average MPS in an organoid during compression is not equal to the compression ratio value used to parameterize injury severity in this study. Our previous publication used finite element simulations to relate the compression ratio to the average MPS.^35^ A compression ratio of 0.3 (which was intermediate in this study) corresponded to an MPS of around 0.15, which is typical of concussive events in American football players.^61^ A compression ratio of 0.5 (which was the upper limit in this study) corresponded to an MPS of 0.3, which caused severe injury in pigs.^62^ The duration of the insult was approximately 30 msec. This pulse duration is in the range observed in human cadaveric tests.^63^ In summary, the range of deformations used to injure organoids spans the full range of deformations that cause injury in patients, from concussive injury on the low end to severe injury on the high end. By contrast, the genetic variants used to increase injury susceptibility represent just a small subset of the genetic factors that could modulate TBI susceptibility in patients.

The *MAPT* mutations studied are rare, and their capacity to exacerbate TBI pathology in patients is unknown. Nevertheless, these results prove that one SNP can exacerbate the phenotypes induced by injury *in vitro,* even though that SNP does not induce those phenotypes in the absence of injury. They provide proof of the concept that this simple, reductionist model can reproduce gene-trauma interactions in a human pre-clinical system. These findings support the hypothesis that patient-specific genetic traits can alter susceptibility to TBI. However, genetic traits are not the only patient-specific factors that influence TBI outcomes. Patient history also plays an important role.

An individual’s vulnerability to TBI depends on their history of TBI, particularly their recent history. This principle is the foundation of clinical practice in the management of concussion, which emphasizes extra precautions against a second impact until the symptoms of the first impact have resolved.^64^ In rodents, the brain is vulnerable to another impact after injury because mitochondrial function is disrupted by injury and resilience normalizes as mitochondria recover.^65^ Mitochondrial function also declines temporarily in humans after injury.^66^ This organoid model reproduces this phenomenon (Fig. 2B), so it could enable systematic investigation of the relationship between vulnerability and mitochondrial dysfunction in a human pre-clinical system.

This model has several important limitations. A total of eight different iPSC lines derived from four different donors were used in this study, which is not enough to represent the genetic diversity of the TBI patient population, but does allow some quantification of the effect of donor-to-donor variation on outcome. This effect was either not statistically significant or minimal across all outcomes. Many important aspects of TBI pathology cannot be reproduced in an organoid model due to the absence of microglia, the blood–brain barrier, and other important players. Whole animal rodent models can reproduce TBI pathologies throughout the brain and beyond.^67^ However, significant species dependence in TBI pathology limits the capacity of rodent models to predict therapeutic success.^68^ An exciting recent development that may ultimately combine the best of rodent and organoid models is the capacity to graft organoids into the rodent brain.^69,70^ The rodent host not only creates an opportunity to measure systemic effects but also promotes more mature anatomy and electrophysiological function in the grafted organoid.^71^ Following organoid grafting into a rodent brain, it would be relatively straightforward to add TBI using the well-established controlled cortical impact^72^ or fluid percussion^73^ models.

In conclusion, the acceleration of neurodegeneration by TBI is an important public health challenge. A biofidelic mechanical insult applied to a human cortical organoid can reproduce important components of post-traumatic neurodegeneration *in vitro*, including genotype-dependent pathology. It can also derive a scientific opportunity from this challenge because injury induces tau oligomerization on an experimentally practical timescale in human cortical organoids without genetic manipulation.

## Transparency, Rigor, and Reproducibility

This study was preregistered on the Open Science Framework (OSF, https://doi.org/10.17605/OSF.IO/RNGM3).

One healthy iPSC line and eight human iPSC isogenic lines were used (two tau mutations and their matched isogenic controls), with up to 24 cortical organoids per line divided across sham, mild (25%), and severe (50%) trauma groups. Injuries were performed in random order, and organoids with abnormal morphology were excluded based on pre-test imaging.

Data analysis, including image quantification and Western blotting, was conducted by personnel blinded to group assignments. Measured outcomes included mitochondrial and viability staining, tau and phospho-tau levels, neurodegeneration markers (LDH, NFL), and immunostaining for MAP2, CP13, and T22. ANOVA was used to assess main and interaction effects of genotype and trauma, followed by Tukey *post hoc* testing. Sample size was based on feasibility and prior success in similar organoid studies. All protocols and data will be made available in a public repository upon publication.
