# Supplementary material for: A Model of Traumatic Brain Injury Oligomerizes Tau in Cortical Organoids and Induces Clinically Relevant Pathologies that Synergize with MAPT Mutation
Source: J Neurotrauma. 2025 Nov 13;42(21-22):2004–18. doi: 10.1177/08977151251374286 (PMC12836475; doi:10.1177/08977151251374286)
Supplement: Supplementary file 1 [file 08977151251374286_supplementary_data.docx]

**Supplementary data**

| **qPCR Primers** | | |
| --- | --- | --- |
| **Primer** | **Forward Sequence** | **Reverse Sequence** |
| GAPDH | GAACGGGAAGCTTGTCATCAA | ATCGCCCCACTTGATTTTGG |
| COL1A2 | CCTGGTGCTAAAGGAGAAAGAGG | ATCACCACGACTTCCAGCAGGA |
| FOXG1 | GCCAGCAGCACTTTGAGTTA | TGAGTCAACACGGAGCTGTA |
| PAX6 | CCCCACATATGCAGACACACA | GAACTGACACACCAGGGGAAA |

**Table S1. Forward and reverse primers used for RNA isolation in qPCR**

**Figure S1. Quality control for isogenic organoids.** (A) Growth curves of organoids, n = 8 lines, n =3-5 organoids averaged across 3 plates. (B) Table of lines. Rows of the table that are the same color describe lines that were derived from the same donor. (C) Graphs of 20-day organoid gene expression by qPCR for forebrain progenitor markers (PAX6 and FOXG1) demonstrate that all 8 lines pass QC metrics. Dotted line illustrates QC thresholds. Negative QC marker COL1A2 denotes lack of mesoendodermal off-target cells. n = 3 plates per line, n = 3 organoids pooled collected randomly across the plate. (D) Representative images of 2-month organoid sections stained for pan neuronal marker MAP2 and deep layer cortical marker CTIP2. (scale bar = 200 µm)

**Figure S2. Immunostaining for 60 and 180 day old WTC organoids.** Representative images of (A) day 60 and (B) day 180 WTC-11 cortical organoids expressing MAP2 (neurons), S100B and GFAP (astrocytes) and SOX2 (neural progenitors). Scale bar = 50 µm, n=3 organoids.

**Figure S3. Western blots showing CP13, PHF1, total tau and actin in 4-month-old organoids from all cell lines.**

**Figure S4. Immunostaining for SSEA and OCT in WTC-11 iPSCs** Representative images of WTC-11 hiPSCs cultured for 3 days (n=3 wells, scale bar= 50 µm).
